# Editorial: Insights in computational genomics: 2025

**DOI:** 10.3389/fgene.2026.1995803

**Published:** 2026-10-07

**Authors:** Yizhuo Yang, Zhibin Lv, Rosalba Giugno, Quan Zou

**Affiliations:** 1 AiBioChem Lab, College of Biomedical Engineering, Sichuan University, Chengdu, China; 2 College of Biomass Science and Engineering, Sichuan University, Chengdu, China; 3 Universita degli Studi di Verona, Verona, Italy; 4 University of Electronic Science and Technology of China, Chengdu, China

**Keywords:** biological priors, biological sequence analysis, comparative genomics (CG), genomic data infrastructure, long-sequence modeling, protein language models (PLMs)

## 1 Introduction

DNA, RNA, and protein sequences are the fundamental carriers of biological information. High-throughput sequencing, long-read technologies, and large-scale genomic projects continue to expand both the volume and diversity of data available for computational analysis. In parallel, machine learning, pretrained language models, comparative genomics, and theory-driven inference methods are broadening the range of biological questions that can be addressed directly from sequence and genomic information. Today, the central challenge is no longer simply whether a predictive model can be built but also how to construct suitable sequence representations for a given task, how dataset construction biases affect generalization, and whether computational outputs correspond to structures or quantities with clear biological meaning.

The seven contributions collected here follow three related lines of inquiry. DFL-MHC, microbial optimal growth temperature prediction, HFTC, and HyenaCircle examine sequence representation and predictive modeling in proteins, proteomes, fungal ITS sequences, and long eccDNAs. Tspecies and the comparative genomics study of *Micrococcus* luteus turn to inference and interpretation under biological constraints; HFTC also links these two areas by explicitly incorporating taxonomic hierarchy. A Mini Review of public human genomic data infrastructures brings architecture, governance, and interoperability into the same discussion. Read together, these studies make the boundaries between model representation, biological validity, and data reliability increasingly difficult to separate.

## 2 Sequence representation and predictive modeling

Predictive modeling of biological sequences starts with sequence encoding. Li et al. proposed DFL-MHC, which integrates ESM-1b and ESM-2 representations with multi-view sequence encoding, BiLSTM, and multi-head attention for MHC protein identification. Hand-crafted descriptors, such as AAC, DDE, and CTD, yielded independent-test accuracies of approximately 0.905–0.913. ESM representations alone exceeded 0.95, while the full DFL-MHC model reached an accuracy of 0.9727 and an MCC of 0.9457. The advantage of pretrained protein representations is evident. Feature count alone, however, says little about how much of that information is useful.

The initial fused multi-model representation was 5,120-dimensional. PCA reduced it to 224 dimensions, after which performance improved on both the training and test sets relative to the uncompressed features. At the representation level, before application of the final BiLSTM-attention classifier, the dual-view, dual-PLM features achieved a test accuracy of 0.9689. Two views generally outperformed one. Adding a third view (the central segment) did not produce consistent gains. The pretrained models supplied a richer pool of candidate information, but effective use of that pool still depended on reducing redundancy and matching the scale of the information to the task.

Dlakić and Inskeep reported a complementary pattern in microbial optimal growth temperature (OGT) prediction. They compared 20-dimensional amino acid frequencies, 400-dimensional dipeptide frequencies, and 1,024-dimensional ProtT5 proteome embeddings across several regression models. The best NN-PLM model achieved a test RMSE of 2.94 and an *R* ^2^ of 0.91. The corresponding values for the dipeptide-based neural network were 3.24 and 0.89, respectively. This contrast suggests that representation quality and downstream model capacity contribute together rather than substituting for one another. Cost changes the picture. Computing ProtT5 embeddings takes minutes, whereas dipeptide-based neural network predictions take seconds. At scale, that difference matters.

Wang et al. proposed HFTC. Their study approached fungal internal transcribed spacer (ITS) classification as an information compression problem. Starting with UNITE data, the authors removed ambiguous or low-quality records and built a training set of 251,630 sequences representing 25,163 species. The representation combined bidirectional k-mers, Word2Vec, and average pooling; at k = 7, this reduced the conventional frequency representation from 16,384 to 200 dimensions. On Test10, HFTC achieved a species-level accuracy of 0.9525 and a hierarchical accuracy of 0.9509, with an inference time of 0.37 ms per sequence. This substantial dimensionality reduction did not come at the expense of task performance, suggesting that much of the discarded dimensionality was not essential for this classification task.

HFTC also showed that the preferred information granularity changes with biological resolution. Higher taxonomic levels often favored longer k-mers; the majority of species-level classifiers performed best at k = 7. Average pooling, however, discards positional information, so local motifs or subsequences relevant to distinguishing closely related taxa may be underrepresented. What, then, should be compressed? The answer depends on which sequence information the biological decision actually requires.

HyenaCircle, developed by Li et al., sits at the other end of contextual modeling, targeting long eccDNA prediction in the 1–5 kb range. From 45,355 FLED detection results, the researchers retained 23,812 positive sequences and matched them with 20,000 negative sequences from regions with low eccDNA detection. After adding 100 bp of genomic flanking sequence around each breakpoint, they used HyenaDNA for long-range, ordered nucleotide context modeling, achieving an AUROC of 0.715 and a recall of 0.776. Truncating the flanking region caused only limited AUROC changes, accompanied by trade-offs between sensitivity and specificity, suggesting that longer context does not automatically translate into more useful information.

HFTC and HyenaCircle adopt different information strategies—the former compresses local patterns early, while the latter preserves long-range order before pooling—yet both converge on the same principle: effective representations are selective, not maximal. At the same time, all three studies show that part of the performance ceiling is already determined before training begins. HFTC cleaned ambiguous records, restricted over-represented Species Hypotheses, and used five independent test sets with no overlap with the training sequences. The OGT study removed redundant strains of the same species to reduce data leakage; however, mesophiles still accounted for 90.8% of the final data, which is an underrepresentation of extremophiles. HyenaCircle’s positive labels came from FLED long-read detections, where structural variation, alignment errors, low abundance, and sequencing depth can all affect label reliability. While these are not all instances of “data imbalance” in the same sense, together they illustrate that sampling, redundancy, annotation, and upstream detection determine what information a model can actually acquire. Thus, dataset construction is itself part of the computational method.

## 3 Biologically constrained inference and genomic interpretation

High statistical performance is fully meaningful only when outputs remain consistent with real biological entities. HFTC uses hierarchical accuracy to evaluate the complete taxonomic path, rather than relying solely on terminal species labels. Differences in representations and architectures across classifiers mean that differences in hierarchical accuracy cannot be attributed to any single component. However, the comparison still shows that species-level accuracy alone does not fully describe whether predictions remain consistent across the entire taxonomic tree. When biological structure is part of the task itself, evaluation metrics should reflect that structure.

Li et al. proposed Tspecies to correct Ks-based estimates of species divergence times. Traditional Ks-based dating commonly estimates divergence time using mean Ks and a substitution rate. However, coalescent theory indicates that average gene divergence can predate species divergence by approximately 2Ne generations—ignoring ancestral effective population size thus leads to systematic overestimation. The authors performed simulations using ms and seq-gen across varying Ne, divergence times, and 23 substitution rate levels, generating 10,000 pairs of 1,000-bp homologous sequences per condition, and used a generalized additive model to describe the relationship between Ne and Ks variance. The reported *R* ^2^ = 0.997 refers to the goodness-of-fit of this simulated relationship, not the *R* ^2^ for actual species divergence time predictions.

In the *Liriodendron* case study, Tspecies estimated the divergence between the North American and eastern Chinese lineages at 1.44 Ma under a 30-year generation time assumption, closely matching the BPP result of 1.38 Ma based on a coalescent model. In contrast, the direct mean-Ks approach yielded a result of 4.36 Ma. While 1.44 Ma is not a directly observable historical truth, this result demonstrates that explicitly introducing a population-genetic model can substantially alter the interpretation of sequence divergence data. HFTC and Tspecies thus use biological priors in different ways: the former constrains the prediction structure with taxonomic hierarchies, and the latter corrects the meaning of an inferred quantity with coalescent theory. The common prerequisite is that the prior assumptions and their scope of applicability must be clearly stated.

Kabiraj et al. took the discussion a step further: what do genomic differences actually mean? Through the comparative genomics of *Micrococcus luteus*, the authors examined 100 high-quality genomes and selected 12 strains from six isolation sources for a more detailed comparison. Genome sizes remained largely within the range of ∼2.4–2.8 Mb. Genomic islands, insertion sequences, prophages, and other variable elements showed greater variation. Each strain contained 21–37 genomic islands and 28–76 insertion sequence elements. Notably, AS2, an industrial wastewater isolate, carried a relatively large accessory genome and more metal-resistance-related genes. This pattern is consistent with a possible role for mobile and accessory genetic elements in environmental adaptation.

The authors were careful not to turn this association into a definitive mechanism. Clear ecological-niche-specific gene preferences appeared in only a few cases, while many hypothetical proteins, unclassified KEGG orthologs, and DUF domains still require computational and experimental validation. Database annotations can suggest potential functions. They cannot, by themselves, demonstrate phenotype or mechanism. Across HFTC, Tspecies, and *M. luteus*, the emphasis therefore shifts. Numerical performance is only a starting point; the harder question is what biological claim the evidence can actually sustain.

## 4 Genomic data infrastructures and responsible reuse

The reliability of computational genomics is also shaped by a practical question: can the necessary data actually be accessed, combined, and governed at scale? Tangaro et al. examined public human genomic data infrastructures along three axes: architecture, governance and access control, and interoperability. Centralized repositories such as EGA and dbGaP provide established systems for curation and controlled access. Cloud-native platforms move computation closer to large genomic datasets. Federated infrastructures take another route: the data can remain at institutional or national nodes. In practice, these arrangements are often blended. Scalability, cost, data sovereignty, privacy, and governance rarely point toward the same architectural choice.

Federation can reduce the cross-border movement of raw genomic data. Coordination is still necessary. The GDPR and the EHDS define part of the regulatory setting, while GA4GH mechanisms such as the Data Use Ontology and Passports encode permitted data uses, access conditions, and researcher authorization claims in portable, machine-readable forms. Technical authorization, however, does not settle accountability by itself. Technical connectivity is only the first step toward interoperability. Disease ontologies, sample identifiers, and informed-consent terms may carry different meanings across nodes; when this happens, connected datasets can remain analytically incompatible. The work of harmonization and standardization, therefore, moves across the federation instead of disappearing.

The infrastructure discussion also reaches back to the modeling studies. HFTC depends on the quality and representativeness of its reference taxonomic databases. OGT prediction remains limited by the scarcity of extremophile samples. HyenaCircle depends on computationally derived detection labels. Tangaro et al. add another layer: technical standards and governance influence which data are collected, discovered, accessed, and eventually combined. More samples alone cannot resolve uncertain labels, systematic underrepresentation, or incompatible metadata. A capable model can still be constrained by the data environment in which it operates.

What, then, do these contributions suggest for computational genomics? They do not make a simple case for ever-larger models. Pretrained and long-context representations can improve prediction, but their value changes with the task, the amount of redundant information, computational cost, and data bias. Biological priors can sharpen inference, provided that their assumptions are stated clearly. Comparative genomics offers a word of caution: annotation can support a functional hypothesis, but it is not proof of mechanism. Genomic data infrastructures set another boundary by shaping what can be discovered, combined, and reused responsibly.

The common lesson is less tidy than a single methodological rule. Sequence representation has to fit the biological task. Inference has to preserve biological meaning. The data supporting both must be reliable enough to sustain them. When these conditions align, the growing volume of sequence and genomic data can support conclusions that are accurate, interpretable, verifiable, and biologically meaningful.

